# 类器官在肺癌精准治疗中的应用

**DOI:** 10.3779/j.issn.1009-3419.2020.101.20

**Published:** 2020-07-20

**Authors:** 梓淇 贾, 乃新 梁, 单青 李

**Affiliations:** 1 100730 北京，中国医学科学院北京协和医院胸外科 Department of Thoracic Surgery, Peking Union Medical College Hospital, Peking Union Medical College and Chinese Academy of Medical Sciences, Beijing 100730, China; 2 100005 北京，北京协和医学院八年制临床医学专业 Peking Union Medical College, Eight-year MD Program, Beijing 100005, China

**Keywords:** 肺肿瘤, 类器官, 精准治疗, Lung neoplasms, Organoids, Precision medicine

## Abstract

肺癌的全程管理需要依靠精准医学来完成。肿瘤类器官（patient-derived organoids, PDO）模型的提出，在肺癌现有的以病理和基因等多组学检测结果为基础的精准医学决策体系之外，增加了从体外功能学模型角度出发的“黑箱”决策体系，未来有望填补分子病理指导下的难治性肺癌的治疗方案的空缺。将PDO应用于临床决策，需要验证肺癌PDO与原始癌灶多维度的一致性，更需要验证与临床试验中患者对治疗反应与PDO药敏结果的一致性。本文将对PDO模型作一简介，并在现有的肺癌精准治疗背景下，对PDO的应用场景进行综述。

## Abstract (en)

Precision medicine is an approach to rational treatment selection in the overall management of lung cancer nowadays. The introduction of the patient-derived organoid (PDO) model has established the "black-box" decision-making system from the perspective of *in-vitro* functional models. This may assist as a complement to the treatment selection strategy based on gene-drug correlation. Further validation must be done in multi-dimensional characteristics recapitulation of the primary tumor in organoids and in large-scale randomized controlled clinical trials. This article will give an introduction to the organoid model and review the application scenarios of organoids in the context of the precise treatment of existing lung cancer.

在中国，2015年肺癌总发病人数约为733, 300例，总死亡例数约为610, 200例^[1]^，为发病率和死亡率最高的肿瘤。尽管靶向治疗和免疫治疗相关的新药研发提高了肺癌的总体生存期（overall survival, OS），但2012年-2015年中国的肺癌患者5年存活率仍仅为19.7%，其中农村地区患者5年生存率仅为15.4%^[2]^，而预计肺癌仍会是未来十年死亡率最高的肿瘤之一。导致其预后不良的一个关键原因是新药研发成功率低。与血液系统恶性肿瘤新药研发的Ⅲ期临床试验成功率可以超过50%相比，肺癌该成功率仅为30%^[3]^。面对这一现状，寻找适于肺癌新药研究的临床前模型显得尤为重要。肺癌肿瘤类器官（patient-derived organoids, PDO）作为能够浓缩原始肿瘤的多组学特征、模拟患者肿瘤药敏特性的功能学模型在新药研发过程中脱颖而出。本文将对PDO模型在肺癌精准治疗中的应用背景、现状及前景作一综述。

## 1 PDO概述

### 1.1 PDO定义

PDO是通过对肿瘤组织进行三维培养得到的保留原始肿瘤多项特征的多细胞团^[4]^。经研究证实，结直肠癌^[5, 6]^、子宫内膜癌^[6]^、胰腺癌^[7]^、前列腺癌^[8]^、膀胱癌^[9]^、乳腺癌^[10]^、卵巢癌^[11]^、头颈癌^[12]^以及最新研究的肺癌^[13, 14]^等PDO在基因组、转录组以及免疫组化染色特点上浓缩了原始肿瘤的特征，即二者在多组学层面上重合度高，且基因组中未重合部分为临床意义不明或不可靶向的基因^[5]^。在功能学方面，与原始肿瘤组织高度一致的药敏特性使其成为癌症新药研发的热点模型^[7]^，同时也成为精准医学方案中的潜在新策略。

### 1.2 类器官在指导临床个体化用药方面的优势

目前应用于肿瘤研究的临床前模型包括细胞系、二维肿瘤细胞培养（two-dimensional cancer culture, 2D-CC）和小鼠移植瘤模型^[15]^。细胞系在肿瘤研究中应用最早且最为广泛，其优势在于可操纵、周期短、通量高^[16]^，利于构建具有某种基因突变的肿瘤细胞模型，以研究基因-药物敏感性关系。但同时，其劣势在于由于其细胞成分单一，难以描述肿瘤的复杂性（包括瘤内及瘤间异质性）^[17]^，即难以反映不同患者肿瘤的个体化特征，无法用于指导临床个体化用药。2D-CC模型取材自患者肿瘤，可以保留原始肿瘤的异质性，但难以经历多次传代建库，其中2D肺癌细胞模型（two-dimensional lung cancer, 2D-LC）在经过5次传代后生长速率会明显减慢，细胞开始衰老。小鼠移植瘤模型则能很好地保留原始肿瘤的结构、细胞组成、分子生物学特征和原始肿瘤的复杂性^[18]^，并且最多可以经过14次传代后仍在组织学、基因组上保持稳定^[14]^。但是移植瘤模型培养周期在2个月-10个月^[18, 19]^，成功率平均在23%-90%^[20]^，不适于指导临床个体化用药和高通量药物敏感性筛选。

相比之下，PDO具有以下优势：①PDO可以很好地代表并浓缩原始肿瘤的诸多形态学及分子生物学特征，且能部分模拟原始肿瘤的生理和药敏特性^[5-10, 14, 21, 22]^；②PDO易于构建，目前可以通过肿瘤手术标本、穿刺标本及循环肿瘤细胞^[23]^建立PDO模型；③PDO的增殖速度较快、培养周期短、培养成功率较高，应用于临床时药敏结果回报周期短^[24]^；④PDO基因及生理特征稳定性好，经过多代培养仍保持稳定^[4]^，可用于构建PDO生物样本库^[22, 25, 26]^。从指导未来精准治疗药物研发角度来讲：①PDO可操作性好，实验室可以对PDO模型进行基因编辑等操作^[27]^；②PDO可以进行高通量药敏筛查，可以提高药物筛选效率^[28]^；③其包含多种细胞类型，可以在简化环境下研究肿瘤微环境中某些细胞间相互作用和形态学发生过程^[4, 29, 30]^；④可用于构建疾病发生模型^[27]^。相比之下，PDO是一种经济-时间费效比低且未来应用广泛的临床前模型。

### 1.3 肺癌类器官的成功培养

肺癌在不同个体间体现出的巨大异质性体现在肿瘤的临床特征、组织学特征、多组学检测结果以及药物敏感性上^[17]^，驱动着肺癌的治疗走上个体化、精准化路线。然而由于个体间基因检测结果在丰度、驱动基因、伴随基因等方面异质性大，在现有体系下仅根据驱动基因选择治疗难以实现每例患者的真正精准个体化。而如前所述肺癌PDO相较于其他临床前模型临床使用价值更高，故培养肺癌PDO势在必行。肺癌有诸多组织学亚型，目前腺癌、鳞癌、腺鳞癌、大细胞癌、小细胞癌均能被成功培养^[13, 31]^。经短期培养的肺癌类器官（short-term lung cancer organoid, stLCO）指培养1个月-3个月获得的肺癌类器官；经长期培养得到的类器官（long-term organoid, ltLCO）指培养时间超过3个月的类器官，往往可以以不小于1:3的比例传代超过10次，且不存在随着传代次数增加而传代效率降低的现象^[14]^。ltLCO可以经受长于1年的低温储存，并在冻存后可以成功复苏。

成功培养出的肺癌PDO能够在组织学结构上与原始肿瘤保持一致，其中腺癌的诸多病理亚型，包括贴壁、腺泡、实性、乳头和微乳头等病理学特征也能够在肺癌PDO中保持一致^[14]^。①遗传学特征方面：ltLCO可以在多次传代（> 10次）后仍保留原肿瘤的主要突变和基因拷贝数变异（copy number variations, CNV）^[13, 14]^。肺癌PDO对药物的敏感性也遵循“突变-药物”关系^[32]^，如携带乳腺癌2号基因（breast cancer 2, *BRCA2*）突变的肺癌PDO对奥拉帕尼敏感，携带表皮生长因子受体（epidermal growth factor receptor, *EGFR*）突变的肺癌PDO对厄洛替尼敏感^[13, 14]^，携带*EGFR*突变/肝细胞生长因子受体扩增突变的肺癌PDO对克唑替尼敏感^[13]^；②新药方面：携带人表皮生长因子受体2（human epidermal growth factor receptor 2, *HER2*）突变的肺癌PDO对新药吡咯替尼敏感，患者用药后也获得了部分缓解（partial response, PR）^[33]^；③联合用药的药敏试验方面：PDO的体外实验在1例患者中证明了MEK/PI3K抑制剂联合成纤维细胞生长因子受体（fibroblast growth factor receptor-1, *FGFR1*）抑制剂在*FGFR1*扩增患者中可能有效，并在活体（*in vivo*）试验中得到了验证^[14]^。尽管stLCO培养时间较短，但已有足够多的细胞可用于药敏试验和生物标志物的临床前试验^[14]^。目前，肺癌PDO从离体培养到药敏测试的平均培养周期是2周，细胞生存曲线往往在用药处理后第6天绘制^[13]^。即：从离体到得到药物敏感性结果的平均周期约3周。在时效性上，有用于临床筛选药物的潜力。

## 2 肺癌精准治疗策略

肺癌精准治疗的现状肺癌精准治疗即利用生物标志物（biomarkers）将患者细分为不同亚型，以筛选出对某一治疗敏感的人群，从而针对该人群精准用药。传统的肿瘤原发灶-淋巴结-转移（tumor-node-metastasis, TNM）分期仅是在肿瘤的进展过程中捕捉了诊断这一瞬间的局部和远处侵犯程度，既无法用于回溯肿瘤的发生，也无法用于个体化预测肿瘤对某一治疗的反应。但TNM分期中的肿瘤大小、是否侵犯胸膜、是否有淋巴结转移及远处转移等作为生物标志物，指导了我们对肿瘤预后的基本判断以及是否选择对患者进行手术等局部治疗等，是进行一切治疗的基础。病理分类亦然，病理切片中不同免疫组化标志物也作为生物标志物，帮助临床医生进一步将患者进行分类。但随着对肿瘤认识的发展，越来越多的肿瘤标志物被发现，一部分标志物直接作为新药研发靶点，而另一部分则用于在新药研发的临床试验中对患者分层以寻找适用人群。据统计，至2019年，预测性生物标志物参与了60%的肿瘤新药研发，而使用生物标志物对入组患者进行分层分析的临床试验占全部肿瘤临床试验的39%，与2010年相比增长了25%，提示精准治疗在肿瘤治疗中占据着越来越重要的地位。总而言之，精准诊断意味着临床医生应该把握两条主线：一条是传统诊断，包括：形态影像学、功能影像学、普通病理学和肿瘤标志物等；另一条则是精准诊断，包括：以中心法则为核心的基因组、转录组、蛋白质表达，甚至表观组学、微生物组学等。两条主线并行，才能够提供足够的决策证据和预后信息（图 1）。其中，处于核心地位且证据等级较高的是通过基因检测结果来选择治疗方案，如根据肿瘤的驱动基因选择靶向治疗方案、根据肿瘤的肿瘤突变负荷（tumor mutational burden, TMB）来指导是否选择免疫治疗等。

**1:**
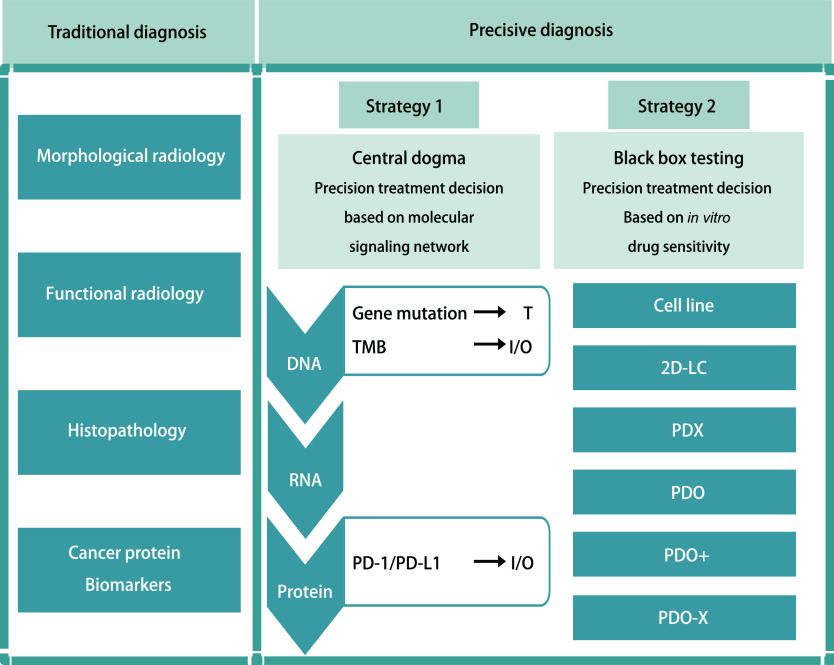
肺癌的精准治疗策略。T：靶向治疗；I/O：免疫治疗；2D-LC：2D肺癌细胞培养；PDX：小鼠移植瘤模型；PDO：类器官；PDO+：与其他组织类型干细胞共培养的类器官；PDO-X：利用PDO构建的小鼠移植瘤模型。 The precision medicine treatment strategy in lung cancer. T: Target therapy; I/O: Immunotherapy; 2D-LC: two-dimensional lung cancer cell culture; PDX: patient-derived xenograft; PDO: patient-derived organoid; PDO+: patient-derived organoid co-culture; PDO-X: xenografts that are derived from patient-derived organoids.

靶向治疗方面，针对*EGFR*、*c-ros*原癌基因1（*c-ros* oncogene 1 receptor kinase, *ROS1*）和间变性淋巴瘤激酶（anaplastic lymphoma kinase, *ALK*）的靶向药物发展迅猛^[34]^，并已经彻底改变了部分肺癌患者的治疗方法。然而，靶向治疗仍有很大局限：①大多数患者仍缺乏可治驱动基因突变，这部分人群目前可以接受化疗、放疗、免疫治疗或联合治疗等，但是患者对何种药物敏感、能否对跨适应证的某药物敏感，单从多组学检测结果判断效果欠佳；②除缺乏可治驱动突变的患者外，有可治驱动突变的患者接受一线治疗后仍有可能出现进展。造成肿瘤进展的原因来自于两方面，一是经一线治疗后肿瘤本身出现耐药突变，二是肿瘤细胞通过产生可塑性逃逸靶向治疗^[34, 35]^。从肿瘤本身产生基因突变以绕过靶点这一耐药机制来讲，并非全部的新发基因突变都已有相应新药被研制上市。而从肿瘤改变可塑性以逃逸靶向治疗这一机制来讲，防止可塑性细胞产生、逆转可塑性、杀伤转化后细胞的药物也仍在研发进程当中^[34]^。此时，根据现有的“多组学精准医学”和“基因-药物关系”、针对新的激活旁路来选择患者的二线用药，在多数情况下颇显困难；③不同患者的伴随突变、胚系基因组的不同^[17]^，给肿瘤细胞提供了不同的基因背景，以现有的分子生物学研究水平，难以完全解读全部患者肿瘤进展的内在原因并做到个体化；④相同突变基因的不同突变类型及突变位点可能导致患者对药物的敏感性不同，如*EGFR* p.L858R突变与*EGFR* 20外显子插入突变对一代*EGFR*酪氨酸激酶抑制剂（tyrosine kinase inhibitor, TKI）敏感性不同，而对同一基因的不同突变类型的研究亦尚不完善；⑤基因的表达和修饰异常也可能对细胞生理学造成较大的影响，而关于转录组、表观遗传组学的研究仍待深入；⑥由于一个基因在分子信号网络中往往位于多个通路的交点，其突变可能对肿瘤的多个信号通路造成影响，从而改变肿瘤的多项生物学特征；⑦对肿瘤的某一通路进行抑制，往往会导致肿瘤通过其他通路激活、下游蛋白异常激活等方式而出现耐药。这些局限成为肺癌治疗中的难点和痛点。

## 3 肺癌类器官在精准治疗中的应用前景

利用PDO指导精准医学是根据“黑箱理论”在药物于肿瘤细胞内具体发挥的分子层面机制未知的前提下，直接根据体外模型对药物的敏感度预测该药物针对患者体内肿瘤的疗效（图 1），因此，可以很好地克服单从基因来推测原肿瘤对药物反应中遇到的上述困难。其在临床上与目前的基于中心法则的分子检测互补，有广阔的应用前景。PDO在肺癌的应用中显得尤为重要。因为目前针对肺癌有效的药物较其他癌种多，故在肺癌的耐药机制的探究以及耐药后二线用药的选择方面，PDO有很大的发挥空间。同时，肺癌的可治靶点相对较多，结合PDO可以在信号通路方面进一步解读肺癌耐药性。另外，因为肺癌的亚型较多，尽管针对腺癌目前以多组学为基础的精准治疗方案显示出诸多优势，但是针对鳞癌、大细胞肺癌、小细胞肺癌等研究仍需PDO助力^[13, 36]^。同时，肺癌样本的获取方式丰富，无论是通过手术获得的大样本、穿刺样本还是胸腔积液都已被用于成功建立器官样培养物，并已验证其驱动突变和药物疗效与原始肿瘤的一致性^[14, 36]^。

然而，只有经过大规模临床研究验证后，体外功能学模型的肿瘤药敏检测结果才能实际应用于临床。在血液恶性肿瘤中，体外功能学模型的培养和药敏检测应用较为成熟，且已经有相关急性粒细胞白血病注册临床试验开展（clinicaltrials.gov identiﬁer NCT: NCT01620216）。实体瘤体外模型的建立相比血液肿瘤更困难，因为其整体培养效率低、培养速度慢等特点，实体瘤的体外模型在临床应用上晚于血液恶性肿瘤。实体瘤中，PDO在胃肠道肿瘤的研究领先于其他肿瘤。已有研究^[28]^验证在转移性胃肠道肿瘤中PDO药物筛选结果与肿瘤分子检测提示的用药选择相符合，且在Ⅰ期/Ⅱ期临床试验中与患者的治疗反应相匹配，阳性预测值为88%，阴性预测值为100%。另一项前瞻性观察性研究^[21]^则证明在结直肠癌中PDO对不同的化疗方案预测能力不同，暗示了大规模临床研究的必要性。目前，只有两项肺癌PDO的临床试验正在进行，但均为单纯的转化性研究，研究对象均为Ⅳ期肺癌，研究目的分别是探究PDO对肺癌标准治疗的预测准确性和判断PDO筛选出的末线治疗手段临床上是否有效（clinicaltrials.gov: TUMOROID, NL49002.031.14; SENSOR, NL50400.031.14）^[37]^。更多关于早期肺癌的验证还有待开展。

PDO的未来发展方向是PDO芯片，其用处之一是提高新药筛选效率。目前光学代谢成像（optical metabolic imaging, OMI）的进展为制作PDO药敏芯片并提高筛选效率提供了可能。肺PDO目前能在96孔板^[38]^和384孔板^[39]^上进行药敏检测，但肺癌PDO芯片仍待进一步研究。

## 4 肺癌类器官在精准治疗应用中面临的问题

PDO与原始肿瘤一致性的探究仍待完善。需要回答的问题包括：癌症PDO模型是否代表肿瘤绝大部分亚群的克隆，抑或PDO是肺癌干细胞群的某一子集的扩增；PDO没有覆盖的原始肿瘤突变是否有明确的临床价值；PDO和原始肿瘤在除基因组外的其他组学研究上是否一致。回答这一问题，需要较大规模的原始肿瘤和PDO分子生物学检测，或建立临床信息完备的PDO生物样本库——非营利性网站HUB（www.hub4organoids.eu）就是其中之一。

临床方面，在PDO与原始肿瘤的一致性研究结果未明前展开大型临床试验可能收效无几，但仍可利用PDO模型研究大型Ⅲ期临床试验中失败药物的原因，并继续展开PDO模型预测临床试验结果一致性的研究。PDO目前应用于临床的另一个壁垒在于培养及药敏检测总周期约3周^[13]^，而利用PDO药物敏感性进行药物筛选的应用需求大多集中于在二线药物或术后辅助治疗的选择上，若想将时间缩短至1周并用于一线药物的选择，仍需较大的科技创新。

PDO的一个关键缺陷在于其培养主要来源于上皮中的干细胞，故缺乏肿瘤微环境细胞参与。随着免疫治疗和抗血管治疗在肺癌治疗体系中地位的不断攀升，研究肿瘤细胞和肿瘤微环境的交互关系显得尤为重要。故需完善与基质细胞、血管干细胞、淋巴细胞等的共培养体系（patient-derived organoid co-culture, PDO+）^[40, 41]^，或利用PDO构建小鼠移植瘤模型（xenografts that are derived from patient-derived organoids, PDO-X）^[40]^。

## 5 结论

随着肺癌在靶向治疗、免疫治疗等方面不断取得新进展，精准治疗在肺癌患者的全程管理中起到越来越重要的作用。然而，由于对肿瘤细胞分子生物学病因研究尚有局限，尤其是对于肿瘤的分子网状信号系统了解甚微，现有的“基因-药物”精准治疗体系仍存在诸多逻辑上待完善的漏洞。相比之下，PDO这一肿瘤体外模型作为功能学模型可以很好地填补这一空缺。肺癌PDO可以通过手术切除组织、穿刺活检、胸水等建立，成为辅助难治性肺癌选药的新兴技术。其能够在基因组、转录组和翻译产物上浓缩原始肿瘤的主要特征。目前肺腺癌、鳞癌、腺鳞癌、大细胞癌、小细胞癌均能够成功培养出PDO。如果肺癌PDO在化疗、靶向治疗及免疫治疗敏感性方面的有效性可以在前瞻性临床研究中得到验证，则其进入临床试验阶段，并进一步用于改善肺癌患者的个性化精准治疗决策方案体系将指日可待。PDO在多癌种中的初步试验数据结果令人鼓舞，并为下一步转化医学的研究提供了切实可行的理论基础。

综上，肺癌分子检测的个体异质性大，PDO可以作为原肿瘤的体外替身，未来帮助实现真正的肺癌超精准个体化治疗选择。
